# Exploratory study of a virtual community for physical activity

**DOI:** 10.1007/s12553-018-0221-y

**Published:** 2018-03-14

**Authors:** Lamia Elloumi, Bert-Jan van Beijnum, Hermie Hermens

**Affiliations:** 1 University of Twente, Enschede, The Netherlands; 2 Roessingh Research and Development, Enschede, The Netherlands

**Keywords:** Exploratory study, Physical activity, Virtual community

## Abstract

Physical inactivity is increasingly becoming part of today’s lifestyle, leading to a rapid increase in the incidence of diseases including cardiovascular disease, diabetes, and obesity. These chronic diseases are, for the most part, preventable by adopting a healthy lifestyle including regular physical activity. To help people maintain appropriate physical activity levels, researchers are developing interventions based on concepts from social science and ICT solutions. In this line, we investigate virtual communities (or social networks) as a candidate solution to support people in achieving their daily physical activity goals. This study observes and explores the differences between using the virtual community and a physical activity monitoring system on the physical activity level. We designed an exploratory study with a duration of 9 weeks in which an intervention group used a virtual community with a physical activity monitoring system and a control group used only a physical activity monitoring system. The results of this exploratory study demonstrate that using virtual communities may motivate and support people in their daily physical activity; in particular, we observed a decrease in the use of the system later than was observed in previous studies. Future investigations are needed to confirm the effect of the virtual community on physical activity.

## Introduction

According to the World Health Organization [1], physical inactivity is the fourth leading risk factor for global mortality, causing an estimated 3.2 million deaths globally. Moderate regular physical activity has significant benefits for health and can reduce the risk of occurrence of cardiovascular disease, diabetes, colon and breast cancer, and depression. Physical activity should not be mistaken for physical exercise. Physical activity is defined as any bodily movement produced by skeletal muscles that requires energy expenditure. Physical activity includes physical exercise but can also involve active transportation, working or household chores, or more general activities of daily living. Although the benefits of regular physical activity are well established, a European survey study reported that only 31% of people are sufficiently physically active.

Social support contributes positively to physical activity behaviour change [2]. In particular, social support from family and friends has been shown to be consistently and positively related to regular physical activity [3]. Social support [4] for physical activity can be instrumental (e.g., the provision of tangible support), informational (e.g., sharing information about the benefits of physical activity), emotional (e.g., calling or messaging to see how the person is doing with a new physical activity plan), or appraisal (e.g., providing encouragement or reinforcement). Existing ICT-based systems, e.g., social networks, are mainly used to provide emotional and informational support for health behaviour change. Many interventions and systems have been developed to help people be more physically active, e.g., apps to promote physical activity [5], active video games [6] and Internet-based physical activity interventions [7, 8]. Although many of these interventions have been successful [9], a decrease in their use is typically observed after a short period [10]. As a basis for solving the compliance problem and providing all different form of social support, the virtual community is a promising ICT-based system for support in daily physical activity.

In our research, we investigate virtual communities for healthy or populations or with chronic diseases or high risks, in which persons need an active lifestyle. In a previous paper [11], we designed the virtual community platform TogetherActive and investigated the usability of the platform. This virtual community platform uses a sensor-based monitoring system to assess physical activity. The virtual community aims to provide all different forms of social support (informational, emotional, instrumental and appraisal support). Our focus is on providing support through functionalities that can be used to share and collaborate on physical activity and physical activity goals and thereby to provide social support with respect to achieving physical activity goals. This is referred to as “instrumental support”. Additional community-related functions are provided as well, including group formation, a leader board, and an overview of the current status of achieving group physical activity goals. We introduce physical activity goals for groups and individuals. We support competition between groups and cooperation among members within a group. Comparison of achievement among members belonging to the same group is also supported. All of these functionalities are included with the goal of to increasing the awareness and the motivation of users. The ultimate goal of the virtual community is to motivate people to be physically active and to maintain their physical activity levels over the long term. The virtual community can be used as a supporting tool for achieving lifestyle changes for health prevention and chronic disease management.

Based on the results of the previous paper, the system was improved, resulting in TogetherActive V2. Using TogetherActive V2, we conducted an exploratory study in which we investigated and explored the use of the virtual community TogetherActive V2 to encourage physical activity with respect to predefined parameters (usability, system usage and physical activity level) and compared these outcomes for two groups (a control group with no virtual community and an intervention group that utilized the virtual community). Within this exploratory study, we restricted the target population to healthy subjects. We randomized 36 subjects into the two groups (intervention and control). The subjects were employees and students at our university who were classified into the category of healthy people. The study lasted 9 weeks. All subjects had access to the basic physical activity monitoring functionalities. In addition, the intervention group had access to the virtual community functionalities. The outcome parameters were physical activity outcomes and the participants’ usage of the platform functionalities.

This paper is organized as follows. Section 2 presents some related work. Section 3 presents an overview of the TogetherActive system. Section 4 describes the methods used in the study and the study design. Section 5 presents the results obtained in the study. Section 6 discusses these results. Finally, section 7 presents the conclusions derived from the study.

## Related work

To reduce physical inactivity and promote this behavioural change, researchers in the fields of social sciences and computer science are collaborating. Social science researchers base their health behavioural change interventions on a number of theories and models from the social sciences [12], e.g., classical learning theory, the transtheoretical model and social support. These interventions are based on face-to-face meetings, and researchers have recently begun implementing these theories in e-coaching systems [13]. Recent findings have shown a robust relationship in which social support from others can be protective for health, and continues to be an active area of research [2]. Therefore, to effectively promote physical activity, interventions should incorporate social support [14].

Computer science researchers have been using information and communications technology (ICT) to provide, extend, and enhance interventions to promote the level of physical activity among healthy people and chronic patients [8, 15]. The interventions address motivation and monitor physical activity with the goal of changing behaviour regarding physical activity. The assessment of physical activity is included in these interventions; it is either self-reported (for example, through the use of e-diaries and questionnaires) or measured automatically and more objectively (for example, through the use of pedometers, actometers, accelerometers and gyroscopes).

Tele-health interventions were developed to encourage people to become more physically active and to monitor their physical activity. Interventions can be based on exercise or walking sessions, face-to-face consulting/group sessions, public campaigns, mail, telephone or computer/web [16]. Overall, these interventions show positive physical activity outcomes. Interventions based on face-to-face counselling or group sessions have proven to be the most effective [16]. Currently, interventions often involve various physical activity assessment devices, e.g., pedometers and accelerometers, in combination with a Smartphone since this allows participants to continuously access their activity data and to receive appropriate feedback any time it is needed. These interventions can be used to support an ongoing healthcare regime or to provide emergency assistance. Examples of these systems are the UbiFit system [17], which encourages individuals to self-monitor their physical activity, the Shakra system [18], which tracks the daily activities of people carrying phones and allows sharing data between friends to increase the motivation for doing physical activities, and ActiveLifestyle [19], which motivates and assists physical exercise in independently living older adults. In addition, there are many examples of commercial support for health/sports monitoring via personal devices (smartphones and music players), including DailyMile [20], RunKeeper [21], Nike+ [22], Adidas miCoach [23], Fitbit [24] and LoseIt [25]. Although many of these interventions have been shown to be successful [9], in some interventions, e.g., in [10], a decrease in use and in compliance with the use of the system provided is noted after a relatively short period (approximately 4 weeks of use).

Additionally, the spread of virtual communities/social networks and e-support groups offer to researchers the ICT support to promote physical activity behaviour change and to provide social support. Some online portals, e.g., WebMD [26], PatientsLikeMe [27] and MedHelp [28] provide emotional and informational support, which is part of the social support needed in physical activity support. On the other hand, some online social networks, e.g., Facebook, can be used as portals to access the collected physical activity data [29–31], thereby providing the emotional and instrumental support that in turn is only a part of the social support needed in physical activity support. Thus, as virtual communities are promising ICT-based solutions for physical activity support, providing all different forms of social support in combination with physical activity assessment is our main goal in the TogetherActive system designed to increase the motivation and compliance of individuals.

## TogetherActive System overview

The TogetherActive system [11] is a virtual community that provides social support to people during their daily physical activities. It supports them in becoming physically active and maintaining an appropriate physical activity level (in number of steps). The appropriate level of activity is captured by the activity goal, which can be set on an individual basis and depends on the personal context.

**Fig. 1:**
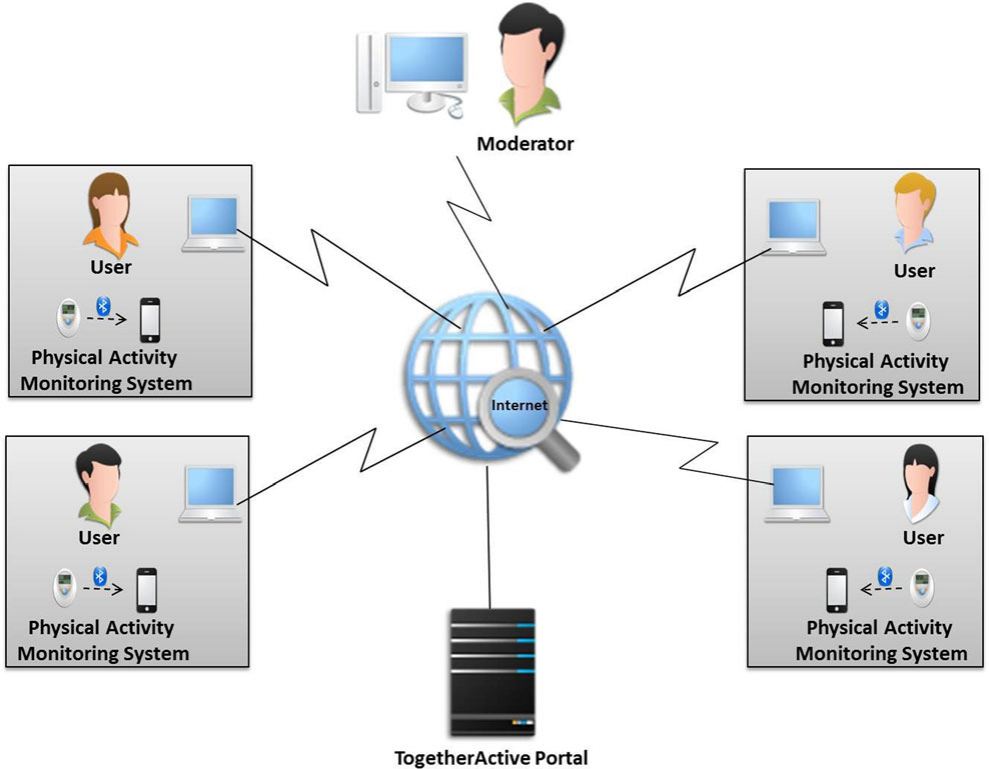
Overview of the architecture of the TogetherActive System [11]

The TogetherActive system (Fig. 1) is composed of a physical activity sensor, a gateway (which can be a smartphone), and a portal. The data collected by the physical activity monitoring system is transmitted from the sensor to the user’s gateway and then synchronized with the portal. The portal is accessible from an Internet-connected device.

The instrumental support is realized by the physical activity monitoring and self-management functionalities. The main functionalities are as follows [11]:

- Self-measuring of the physical activity: A physical activity monitoring system is provided to users to measure their physical activity levels.
- Self-monitoring of the physical activity: Users are able to monitor their physical activity themselves to change their physical activity behaviour.
- Self-comparison of physical activity: Users are able to compare their current physical activity level with previous levels; e.g., the daily level can be compared with the previous day’s level.
- Setting personal goals: The system is about setting physical activity goals. These goals should be realistic and measurable. They are time-targeted, e.g., daily, goals. The users are able to set the physical activity goals themselves.
- Sharing physical activity level with peers: Peers of the same virtual group are able to share their physical activity levels.
- Monitoring the physical activity of others: Peers of the same virtual group are able to monitor each other’s physical activity level
- Setting virtual group goal: A virtual group exists to motivate members who share a common goal. Each virtual group has its own goal.
- Collaboration: Because a virtual group goal is set for each virtual group and this goal is shared among the peers of the virtual group, collaboration (motivating and supporting each other) to reach this virtual group goal is encouraged. The system provides a platform through which the group members can communicate using online or offline messages, see each other’s progress, and provide valuable input and feedback.
- Competition: The TogetherActive community is composed of multiple virtual groups. With each group aiming at achieving its own goal, the system provides a possibility platform for managing and creating competitions between virtual groups.
- Comparison: Within a virtual group, peers can compare their physical activity achievements with those of others and gain insight into the similarities and differences amongst virtual group members.

Appraisal support and feedback is given based on an individual’s physical activity level and achievement compared to his or her personal and group goals. The TogetherActive system provides feedback through its self-monitoring and self-comparison functionalities, and peers in the community can also provide feedback via monitoring of others’ physical activity and through collaboration.

Emotional support is provided by the peers. This is accomplished by publishing discussion on a blog in the TogetherActive community. It is also supported by a synchronous communication service (chat) and an asynchronous communication service (private messaging).

Information support is provided by the system. It is done using published information about physical activity, general information about the pros and cons of physical activities, new facts published about the importance of physical activity, and recommendations about physical activity on a wiki related to the TogetherActive community.

As first step, we conducted a usability study involving 10 healthy subjects [11] to acquire feedback from users and technically improve the system. The outcomes of the usability study suggested a number of system improvements: the portal navigation, the quality of the graphics, the help functionality and the physical activity sensor used. For the new version of Portal TogetherActive V2, we made the improvements suggested in the usability study. We improved the portal navigation, the quality of the graphics and the help menu. Additionally, we included new functionalities: a leader board, an overview of the individual’s current status with respect to achieving the group goal, and other functionalities (these functionalities were designed but not implemented in the previous study [11]). The previous choice for a physical activity monitoring system was the ProMove sensor [32]. The ProMove sensor had some serious usability issues: users found the sensor too large, making it uncomfortable to wear, and the sensor had to be charged daily, which was considered inconvenient. For these reasons, it was decided to use a different physical activity sensor, the Fitbit Zip activity tracker [24], in TogetherActive V2. The outcome measures of ProMove and Fitbit are different. With the ProMove sensor, the resulting IMA values correlate well with energy expenditure for a wide range of activities. With Fitbit, steps are used to quantify the physical activity level, which is limited to activities e.g., hiking, running and climbing. For our purpose within TogetherActive V2, target users will be using the system while working or studying, so walking is their main activity. Thus, using Fitbit coupled with TogetherActive V2 is appropriate. The Fitbit Zip is relatively inexpensive compared to other commercial physical activity trackers, and it has been proven to be a reliable measurement device for measuring steps [33]. Fitbit also offers an API and an SDK, allowing us to collect the raw data from the sensor and use it in our system. With Fitbit Zip, daily charging is not required (it runs on a replaceable 3-V coin battery), and it is a lightweight sensor.

TogetherActive V2 is based on Liferay Portal 6.2 Community Edition bundled with Tomcat. To synchronize the Fitbit data with TogetherActive V2, it is first uploaded to the user’s personal computer. Subsequently, the data are sent via a secured connection (HTTPS) to the Fitbit server and retrieved (also via HTTPS) from the Fitbit server to be placed on the local university server where the portal TogetherActive is hosted. To make it possible to use Fitbit data in the portal, the existing portlets using physical activity data were updated to have the ability to synchronize with Fitbit API. Additionally, new portlets were implemented to provide the new functionalities. Consequently, personal and virtual group goals were updated to be based on the number of steps.

## Methods

### Study design

This exploratory study consisted of a pilot study involving two independent groups: an intervention group and a control group. The study lasted 9 weeks. The participants were randomized into these two groups. The randomization was based on the block randomization method and was performed using an online tool.^1^ When all of the participants had been recruited, the chronological order in which the participants joined the study was used as the input list. The participants in the intervention group were randomized into 4 virtual groups (G1, G2, G3 and G4) for the group-based functionalities provided by the system. The same randomization method was used to define the virtual groups. The input list for the second randomization was based on the resulting order of participants from the previous randomization. Both randomizations were performed by one of the researchers (LE).

#### Participants

Healthy subjects working or studying at the University of Twente who were between 25 and 55 years of age were recruited. For the recruitment process, we used University of Twente Facebook groups and University of Twente mailing lists (MIRA^2^ and CTIT^3^ institutes).

#### Procedures

During the recruitment period, interested subjects received a general description of the study to help them decide whether or not to join the study. The inclusion criteria were: adults who were able to perform daily activity and who were working or studying at the University of Twente. The exclusion criterion was that no partners should participate in the study, which would provide an extra form of support. The subjects who agreed to participate in the study were asked to sign an informed consent form. Given the materials and methods used, no medical ethical approval was required under Dutch regulations. The research was conducted in full compliance with the “Declaration of Helsinki”.

The two groups were invited separately to an introductory meeting. The objective of this meeting was to give the participants within each group an overview of the system and the aims of the study. The participants received a demo of the use of the system and the physical activity sensor Fitbit. They also received credentials that could be used to connect to the system and couple their Fitbits to their profiles. The participants in both groups received a link to a pre-study questionnaire. It was used to collect the characteristics of the participants (more details regarding this point are provided in the subsection “study measures”). After the 9-week study period, the participants returned the Fitbit sensors and received a link to a post-study questionnaire. The control group was asked questions designed to assess the usability of the system, the sensor and the portal functionalities provided to them. The intervention group received the same post-questionnaire plus additional questions regarding the virtual community functionalities.

During the study period, we set 10,000 steps per day as a personal goal for both the intervention and the control groups. This goal has been suggested and accepted as a healthy number of steps to be taken daily by healthy people [34]; hence, this goal is in compliance with the WHO recommendations.

- **Control group**

Participants in the control group received a basic version of the TogetherActive V2 system with the physical activity monitoring system Fitbit. They had no access to the virtual community functionalities.

b. **Intervention group**

Participants in the control group received a full version of the TogetherActive V2 system. After the introductory meeting, they were randomized into 4 virtual groups. This allocation to virtual groups was required to make it possible to use and test the group-based functionalities proposed by the system.

During the study, the participants were asked to collaborate and communicate with participants in their own virtual groups to achieve their daily group goals and to compete against the other virtual groups. The daily group goal was defined as well. Based on the daily steps achieved, the participants received personal points (Table 1); the highest number of points was awarded for achieving personal goals (9 points), and smaller numbers of points were awarded for lower achievement. The group goal was for each member of the group to reach the maximum number of points. Whenever all peers of a group achieved their daily personal goals, every participant was awarded a bonus point (+1). The group goal was the maximum average number of points that could be achieved by a virtual group (10 points). As part of the competition functionality (see Section 3), a leader board was provided to the virtual groups. It displays the ranking of the virtual groups based on their achieved group goals and related points.

**Table 1:** Points attribution

Personal physical activity level	0 Steps	1–4999 Steps	5000–7499 Steps	7500–9999 Steps	≥10,000 Steps	All peers ≥10,000 Steps
Personal Points	0	3	5	7	9	10

### Study measures

The participants completed a questionnaire at the beginning and at the end of the study/intervention. The pre-study questionnaire assessed the baseline measurements and characteristics of the participants, including age, gender, ethnicity, work status (employed, unemployed, studying or retired), education level (basic school, high school, college/university, other), and BMI (<25.0, 25.0–29.9, ≥ 30.0). It also assessed the following:

- Habitual physical activity [35]; resulting in three indexes: work index (to measure physical activity at work), sport index (to measure sport during leisure time) and leisure index (to measure physical activity during leisure time excluding sport)
- Physical activity stages of change [36]; resulting in one of the five stages (precontemplation, contemplation, preparation, action and maintenance)
- Use of social networks in general (e.g., Facebook, Twitter, Google+, Myspace and LinkedIn) and for health or well-being purposes, in particular starting dates of use and how many hours per week were spent on social networking
- Use of monitoring systems/applications (e.g., Runkeeper [21]) for health or well-being purposes
- Basic quality of life (EQ5D) [37]

The post-study questionnaire was supplied in two versions: one for the intervention group and one for the control group. Both versions included a usability questionnaire, questions about the use of the sensor system and questions about the specific functionalities provided to each group. Following the guidelines provided by Lewis [38], the usability results are summarized into 4 factors that are reported as the mean values: overall system usability (OVERALL), system usefulness (SYSUSE), information quality (INFOQUAL) and interface quality (INTERQUAL). The usability questions are based on a 7-point Likert scale (1 for strongly agree and 7 for strongly disagree). After checking the replies from all participants, we decided to exclude from the analysis the replies of one of the participants in the control group, who had replied to all questions with 1 (strongly agree). The questions concerning the sensor included how often the participants forgot to wear or use it, whether they experienced trouble with it, and whether they went on holidays during the period of the study.

Additionally, to obtain insight into the use of the portal, from the portal logs we collected data on the following:

- Total and average number of times the portal was accessed per participant per week (number of sessions)
- Total number of messages exchanged (sent, received and posted) though the functionalities provided by the portal (chat and blogs)

From the system database, we gathered the physical activity (steps) data of all participants in both intervention and control groups. To be able to compare steps data during the period of the study, the baseline week was determined based on an observational analysis of the data.

Based on the step data that were collected, we computed the percentages of days on which specific numbers of steps were taken per day, averaged over the participants of a group (intervention, control, and virtual groups) for the full period of the study and for each week of the study, as follows:

- V1: percentage of days with <1000 steps per day
- V2: percentage of days with 1000–2999 steps per day
- V3: percentage of days with 3000–4999 steps per day
- V4: percentage of days with 5000–7499 steps per day
- V5: percentage of days with 7500–9999 steps per day
- V6: percentage of days with ≥10,000 steps per day

V1 and V2 provide information about sedentary behaviour, and V6 informs about reaching the recommended personal goal for physical activity (10,000 steps per day).

Furthermore, we analysed the differences between the baseline week and the subsequent weeks of the study period for both the control and intervention groups. Additionally, we looked into the group goal achievement (using V6 values for the virtual groups within the intervention group) and the scores and levels reached by all virtual groups.

During the study, we could not control the actual wearing of the sensor, e.g., the times at which it was put on and removed, or the days of its actual use. We needed to clean and adjust the steps data to be able to compare steps data between participants and between groups. According to the manufacturers of the activity trackers, e.g., Fitbit [24], and websites offering physical activity recommendations [39, 40], making 1000 to 3000 steps per day is classified as sedentary behaviour. Therefore, we can consider that a day on which a total of less than 1000 steps were made is a day with no activity. If we assume that a person can wear the sensor for 10 h per day and record a minimum of 1000 steps, we defined a threshold of 100 steps per hour. Based on this threshold, we defined 3 activity levels:

- Hours with no activity (HNA) for hours with 0 steps
- Hours with low activity (HLA) for hours with less than 100 steps
- Hours with activity (HA) for hours with 100 steps or more

We then computed the 3 metrics (HNA, HLA and HA) on the steps data per hour (one hour is from hh:00 to hh:59) for all groups for the entire duration of the study and per week.

Additionally, we took the average number of steps made during hours with activity HA (≥100 steps per hour) per week and excluded the days for which no activity was recorded (HNA = 24). We computed the change in the average number of steps per hour compared to the baseline week as follows:

$$ Change\_ Wee{k}_i= AVG\_ Step{s}_{Wee{k}_i}- AVG\_ Step{s}_{Baseline\_ Wee k}\  with\ i\  in\ \left[ Baseline\_ Wee k+1,9\right] $$

The change value can be 0 steps for an average number of steps per hour for week *i* similar to the baseline week, a positive number of steps for an average number of steps per hour for week *i* more than the baseline week and a negative number of steps for an average number of steps per hour for week *i* less than the baseline week.

To test the statistical significance of the difference between the intervention group and the control group in the change of steps over the weeks compared to the baseline week, we used the two-sample Wilcoxon test, which is also known as the Mann-Whitney test.

Finally, to check for associations between some of the variables (sport index, work index, leisure index, average steps during the period pf the study (taking into consideration hours with activity HA), and total sessions), the correlation analysis method was used. It is test for association between paired samples, using Pearson’s correlation coefficient.

## Results

**Fig. 2:**
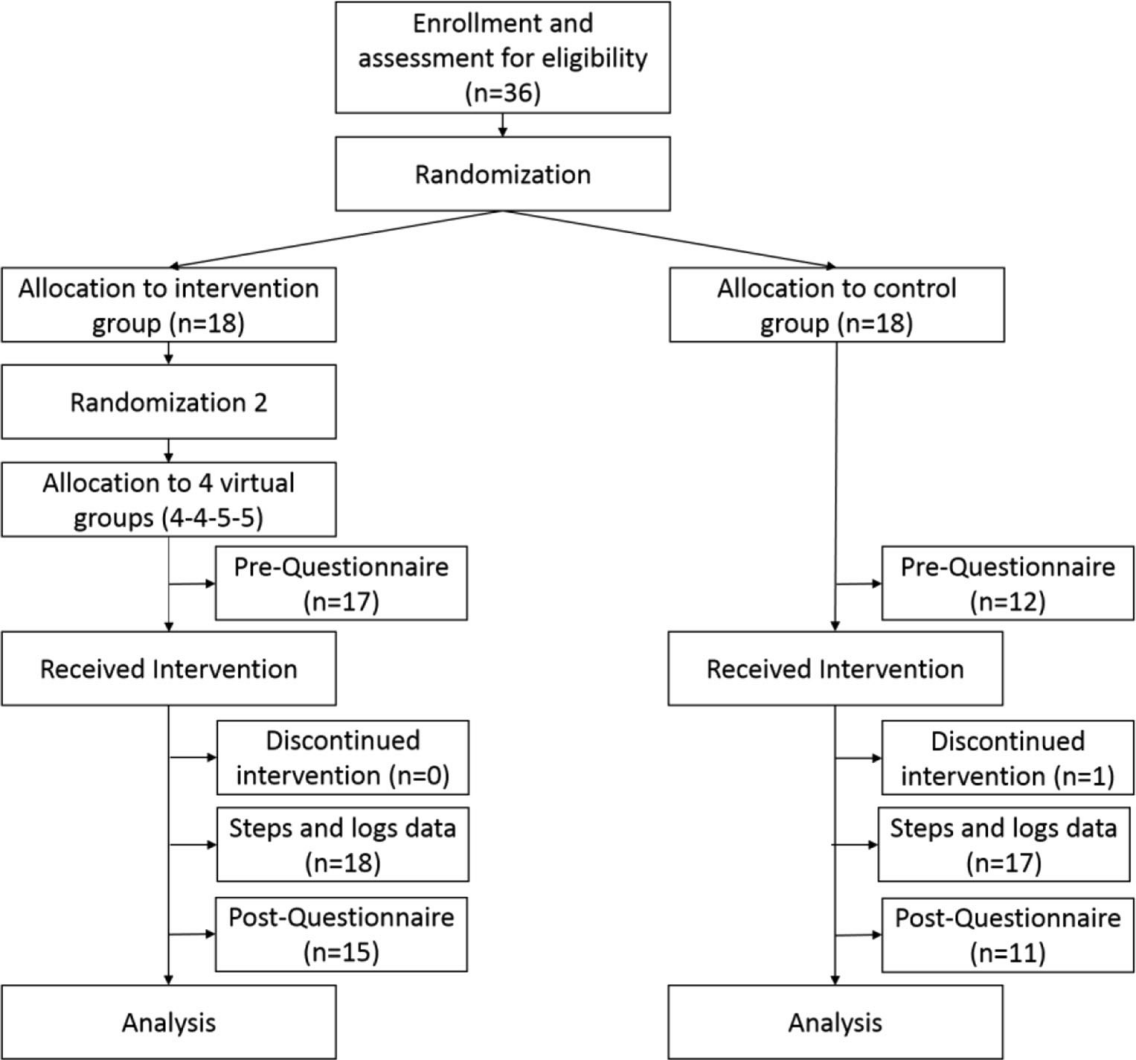
CONSORT diagram of the study design

Figure 2 presents the study design. When recruiting participants for the study, only one person did not satisfy one of the inclusion criteria. Thirty-six participants from the University of Twente were recruited to participate in this study. Through randomization, 18 participants were assigned to the intervention group and 18 participants were assigned to the control group. Thirty-five participants completed the entire study; one participant left the study due to loss of the Fitbit sensor. The pre-study questionnaire was completed by 29 participants (17 from the intervention group and 12 from the control group), and the post-study questionnaire was completed by 26 participants (15 from the intervention group and 11 from the control group). For both questionnaires, we were not able to determine why some of the participants did not respond.

### Demographics

Table 2, which is based on the results of the pre-study questionnaire, presents the demographics of the participants, the use of social networks in general by the participants, and the use of social networks and mobile applications for health or well-being purposes. The results show that the study participants are used to social networks but not to using them for health and well-being purposes. In contrast, almost 60% of the participants used mobile applications for health and well-being purposes, mainly for recording exercise or training or for schedule compliance.

**Table 2:** Participants’ demographics

	Intervention group (*n* = 17)	Control group (*n* = 12)
Gender		
Male	9	4
Female	8	8
Age		
20–29	12	7
30–39	5	4
40–55	0	1
Social Network used		
Facebook	16	11
LinkedIn	14	9
Twitter	4	4
Google+	4	2
Other	1	1
Start of use of social networks		
Less than a month	1	0
1–12 months	0	0
1–4 years	6	5
More than 4 years	10	7
Hours per week for use of social networks		
0–5 h	8	9
6–10 h	7	1
11–20 h	1	1
21–30 h	1	1
Use of social networks for health and well-being purposes		
Yes	0	2
No	17	10
Goal of social networks (for participants by whom it is used for health and well-being purposes)		
Informational	0	2
Medication intake compliance	0	1
Exercise/training schedule compliance	0	1
Exercise/training recording	0	1
Coping with disease	0	1
Other	0	0
Use of applications for health or well-being purposes		
Yes	8	3
No	9	9
Goal of applications (for participants by whom it is used for health and well-being purposes)		
Informational	3	0
Medication intake compliance	0	0
Exercise/training schedule compliance	2	2
Exercise/training recording	5	2
Coping with disease	0	0
Other	3	0

Table 3 gives the health-related indexes of the participants (BMI, habitual physical activity, stage of change and quality of life). The results show that the study participants represent a homogeneous population with respect to BMI index (most of the participants have BMIs between 18.5 and 24.9) and with a good and almost identical quality of life index.

**Table 3:** Health-related indexes

	Intervention group (n = 17)	Control group (n = 12)
BMI		
< 18.5	1	1
18.5–24.9	11	10
25–29.9	5	1
State of change		
Precontemplation	2	0
Contemplation	2	3
Preparation	5	1
Action	0	1
Maintenance	8	7
Quality of Life (EQ-5D) (1 indicates the highest quality of life)		
Mean ± SD	0.91 ± 0.09	0.89 ± 0.15
Habitual physical activity (1 = low; 5 = high)		
Work Index		
Mean ± SD	2.25 ± 0.53	2.06 ± 0.52
Sport Index		
Mean ± SD	1.68 ± 0.92	2.21 ± 0.96
Leisure Index		
Mean ± SD	3.12 ± 0.52	3.17 ± 0.34

### System use and usability

From the post-study questionnaire, we gathered feedback from the participants about the following:

- System usability
- Sensor use
- Specific functionalities provided by the system (taking into account whether the participant belonged to the intervention or the control group)

Table 4 gives a summary of the usability results of the intervention and control groups and the results of the usability study of TogetherActive [11]. After comparing the results obtained with the intervention group with those of the previous usability study of the TogetherActive system, we can conclude that for the TogetherActive V2 system, usability is similar to that of the TogetherActive system, with small improvements in the OVERALL and SYSUSE factors for both the intervention and the control groups. The result shows that the control group scores for the INFOQUAL and INTERQUAL factors of the TogetherActive V2 system are better than those of the intervention group and better than those obtained for the TogetherActive system.

**Table 4:** Usability results for the intervention and control groups and for the usability study of the TogetherActive system [11]

	Intervention group Mean ± SD (*n* = 15)	Control group Mean ± SD (*n* = 10)	Usability study [11] Mean ± SD (*n* = 8)
OVERALL (Q1 to Q19)	3.71 ± 1.51	3.15 ± 1.75	3.81 ± 1.09
SYSUSE (Q1 to Q18)	3.56 ± 1.55	3.02 ± 1.74	3.89 ± 1.03
INFOQUAL (Q9 to Q15)	3.91 ± 1.54	3.38 ± 1.81	3.81 ± 1.06
INTERQUAL (Q16 to Q18)	3.64 ± 1.32	3.03 ± 1.66	3.5 ± 1.29

Regarding the use of the sensor by the participants in the intervention group, one participant never forgot to use it, 10 participants rarely forgot to use it, and 4 participants sometimes forgot to use it. None of the participants experienced trouble with the sensor, and only one participant went on holidays during the study period. Regarding the use of the sensor by the participants in the control group, 2 participants never forgot to wear the sensor, 7 rarely forgot it, one sometimes forgot it, and one often forgot it. Only 2 participants experienced trouble with the sensor. Six participants went on holidays during the study period, but their holidays did not affect their use of the sensor during the period of the study.

Based on the replies to the post-study questionnaire, navigation in the portal was easy in general for the intervention group. However, the intervention group experienced some difficulties in navigation within the pages of a group; it was difficult for them to understand the navigation menu. The aim of the portal and the functionalities proposed (e.g., the leader board, the group goal, group goal achievement and daily steps) were understandable. For the control group, navigation in the portal and the aim and functionalities proposed by the system were easy to understand.

### Portal logs analysis

From the portal logs, information about the number of sessions, the number of pages visited per session and the number of exchanged messages was collected.

During the period of the study, the intervention group accessed the portal twice as often as the control group (approximately 200 sessions for the control group and 430 sessions for the intervention group). Similar to the average number of sessions over the period of the study, the average per week showed that the intervention group accessed the portal weekly twice as often as the control group.

We found no differences between the intervention group and the control group regarding the average number of pages visited per session; in both cases, this varied between 15 to 20 pages per session.

Finally, when looking at the messages exchanged between participants (using the chat and blogs), the participants within the intervention group did not exchange messages; however, two members of one virtual group (G2) posted two messages on the blog of their virtual group’s page.

### Physical activity data analysis

As explained in the Methods section, week 2 is considered the baseline week.

#### Steps per day

Table 5 shows the average percentage of days with a specific number of steps per day (days on which the personal goal was achieved) for the participants in each group for the period of the experiment (the metrics used are introduced in the Methods section).

**Table 5:** Average number of days (in percentage) based on the final number of steps achieved by different groups

Group	V1 (<1000 Steps)	V2 (1000–2999 Steps)	V3 (3000–4999 Steps)	V4 (5000–7499 Steps)	V5 (7500–9999 Steps)	V6 (> = 10,000 Steps)
Virtual Group 1	18.57%	6.56%	3.64%	11.37%	24.90%	34.96%
Virtual Group 2	15.83%	8.12%	10.71%	20.54%	17.05%	27.74%
Virtual Group 3	18.13%	7.74%	8.07%	11.96%	16.18%	37.92%
Virtual Group 4	11.84%	14.69%	14.27%	15.88%	24.74%	18.58%
Intervention Group	16.19%	9.13%	9.20%	15.08%	20.26%	30.14%
Control Group	19.32%	4.19%	9.99%	21.39%	17.87%	27.25%

**Fig. 3:**
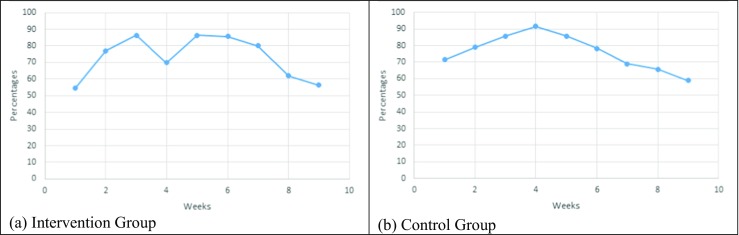
Average number of days (in percentage) with over 3000 steps per day

Figure 3 shows the average value V3 + V4 + V5 + V6 (3000 steps and more) for the participants in each group (intervention, control, and virtual groups) for each week of the study. The data show that, with the exception of week 4, this value increased until week 6 for the intervention group and that it increased until week 4 for the control group.

When looking at V6 for the intervention group over the weeks of the experiment, we observe that it increased until week 6, decreased for the week 7 to 8, and increased during the last week of the experiment (week 9). When looking at the V6 for the control group over the weeks of the experiment, we observe that it increased until week 4, decreased for the weeks 5 to 7, and increased during the last 2 weeks of the experiment (week 8 and 9).

#### Steps per hour

**Fig. 4:**
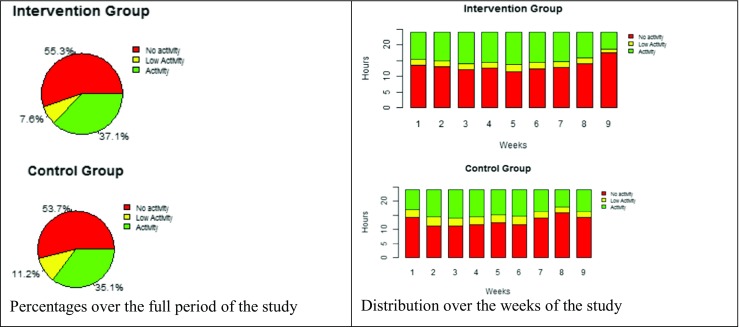
Distributions of the activity levels for the intervention group and the control group

We analysed the participants’ steps data per hour and classified it as hours with no activity (HNA), hours with low activity (HLA) or hours with activity (HA). Fig. 4 presents the percentages of HNA, HLA and HA over the period of the study and the distribution per week for both the intervention and the control groups. The figure gives an overview of the behaviour of the participants with respect to the wearing of the sensor. It supports the assumption that a person wears a physical activity tracker for 10 h on average. In addition, it shows that, based on the group averages, all groups have a similar distribution of activity levels. Over the full period of the study, the intervention group shows 37% activity and 8% low activity, compared to 35% activity and 11% low activity for the control group. For both groups, the activity increases at weeks 4 to 6 and decreases during the last 2 weeks.

**Fig. 5:**
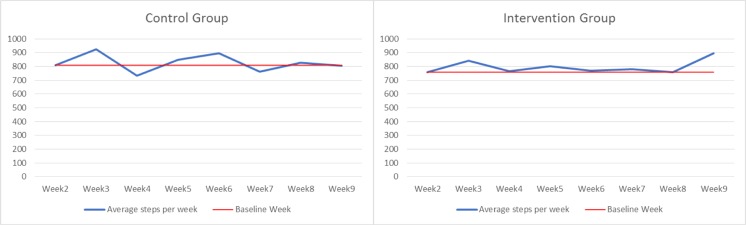
Average steps (HA) per week

Additionally, we considered the average number of steps (only HA) excluding the week with no activity recorded (HNA = 24) for each week of the experiment and computed the change for each week relative to the baseline week (week 2). Table 6 and Fig. 5 show the results for the intervention group and the control group. We observe that for the intervention group, the change is positive for all weeks (except that one week has a change of 0; week 8). The data do not show that the physical activity of the intervention group decreases after 4 weeks. Tabak [6] observed that a decrease in the physical activity of participants in a physical activity intervention occurred after 4 weeks. By the end of the study, we observe an increase in the average number of steps taken by the intervention group. In contrast, the control group has 3 negative changes compared to the baseline week; they occur at weeks 4, 7 and 9.

**Table 6:** Average steps (HA) per week and change in the number of steps (compared to week 2) for each week of the study

	Week2	Week3	Week4	Week5	Week 6	Week7	Week8	Week9
Intervention group	760	843	767	801	771	780	760	897
Control group	809	925	732	848	895	761	827	805
		Change_Week 3	Change_Week 4	Change_Week 5	Change_Week 5	Change_Week 7	Change_Week 8	Change_Week 9
Intervention group		+	+	+	+	+	0	+
Control group		+	–	+	+	–	+	–

### Statistical analysis

To investigate the differences between the intervention group and the control group in the change in the number of steps over the weeks compared to week 2, we used the Mann-Whitney test with the H_0_ hypothesis that both groups have a similar change in the number of steps per week. The computations yielded a p-value of 0.241. The results (small *p*-value) indicate that intervention group displays a significant difference in the change in the number of steps over the weeks comparing to the control group. In fact, the total sample size is seven, the Mann-Whitney test will always give a *P* value greater than 0.05 no matter how much the groups differ.

To check for possible associations between the variables (sport index, work index, leisure index, average number of steps (taking into account only HA) and total sessions), we computed the correlation coefficients with associated *p*-values (Table 7).

**Table 7:** Correlation analysis

Correlations	Control	Intervention
Correlation (sport index, sessions)	−0.06, *p*-value = 0.84	−0.03, *p*-value = 0.89
Correlation (sport index, average steps)	**0.64, p-value = 0.02**	0.2, p-value = 0.44
Correlation (work index, sessions)	−0.39, p-value = 0.20	0.003, p-value = 0.98
Correlation (work index, average steps)	0.26, p-value = 0.41	−0.09, p-value = 0.70
Correlation (leisure index, sessions)	0.22, p-value = 0.48	**−0.56, p-value = 0.01**
Correlation (leisure index, average steps)	0.15, p-value = 0.62	**−0.42, p-value = 0.08**

(Strength of the association: Small (0.1 to 0.3 or −0.1 to −0.3) Medium (0.3 to 0.5 or −0.3 to −.5) and Large (0.5 to 1.0 or −0.5 to −1.0)).

We observe that in the intervention group there are three strong associations (as shown in bold in Table 7): positive strong association between sport index and average steps for control group, and two negative strong associations between leisure index and sessions, and between leisure index and average steps for intervention group.

For the control group, the strong association means that being active and doing a lot of sport during leisure time was associated with an increased average number of steps during the period of the study. This finding is shown in the sport indexes for both groups (2.21 for control group vs. 1.69 for intervention group, Table 3).

For the intervention group, the strong association mean that being physically active during leisure time was associated with a decreased access to the system and decreased average number of steps. This finding was not observed with the control group although both group had similar leisure indexes (3.17 for control group vs. 3.12 for intervention group, Table 3). This can contributes to the influence of the TogetherActive V2 system and proposed social functionalities comparing to a traditional system with only physical activity monitoring functionalities.

Although medium associations exist, we cannot draw observations since related *p*-values indicate weak correlations.

## Discussion

The objective of this study was to explore the use of the TogetherActive V2 physical activity platform with respect to a predefined set of outcome parameters and to compare these outcomes for an intervention group and a control group. The main categories of outcomes considered are the usability of the system, system usage and physical activity outcome measures.

Regarding the usability, TogetherActive V2 showed an improvement in usability results for the overall system usability and the system usefulness factors compared to TogetherActive [11]. The participants had the opportunity to comment on the usability of the new system, but none of the comments gave extra insight into the usability results. Based on the results of the sensor use questionnaire, the study participants were satisfied with the sensor used and declared compliance regarding wearing it. Based on the customized questionnaire about specific functionalities provided by the portal, the participants had difficulty with navigation within the portal and in understanding the primary aim of the system. Concerning the implemented portlets, the participants had no difficulty understanding or using the offered functionalities and rated them positively. The system should be improved to make it more intuitive and to solve the difficulties that were reported by the participants. Improvements can be made in the design of the pages of TogetherActive V2 and in navigation between the pages. With these future technical improvements, a new usability study based on a task-oriented approach would improve the usability outcome factors of the system.

The use of the system was analysed using the portal logs. The intervention group using TogetherActive V2) had access to a number of additional functionalities that were not available to the control group. The TogetherActive V2 platform included basic community functionalities, e.g., monitoring of the peers’ physical activity data, making it possible to take part in competitions between virtual groups, and online and offline communication (through chat and blogs). The results showed that the intervention group accessed (and therefore used) the portal more often than the control group. Although the results showed that the participants accessed the portal, they did not interact with each other through the portal (no messages were exchanged and no communication was established between the participants). Although the required functionalities were provided, the communication modes were limited, and no notifications mechanism was provided for notifying other members when a participant received a message from other participants or from the system or about recent achievements. The system needs to support a notifications mechanism to make the communication more interactive. This notification system could be provided by an application running on the smartphones of the participants, or even on smartwatches, if these are used by the participants. Such a notification system can be provided by connecting the system to existing third parties, e.g., the Google cloud messaging service, as a way to send notifications to the mobile device. Additionally, it is important to invest on how we can start, activate and moderate the dynamism of interactions between the virtual community’s peers and virtual groups. One candidate option is the introduction of roles, e.g., choosing a moderator to initiate and monitor the interactions. Another option would be the exploration of social activation and enhancement of the involvement of peers and their interactions via a notification mechanism that targets the peers more than the concerned person. With this approach, we can transform the physical activities that each participant needs to perform into social activities involving physical activities that are suggested by the peers and mediated by the system.

With respect to the sensor and physical activity outcome measure, in the current study, we did not ensure the participants’ compliance with wearing the sensor, and we could not control the wearing behaviour (starting and ending time of wearing or days of not wearing sensors). The validity of the measured data (steps data) was a real issue that required additional attention during the processing the data.

To be able to explore the steps data obtained from the intervention group and control group in greater depth or even between peers, we needed a fair measure that takes into consideration starting and ending, the duration and the intensity of the real physical activity, in addition to the time of wearing and removing the sensor. In the analysis, we defined hours with no activity (HNA), hours with low activity (HLA) and hours with activity (HA), and we considered only the HA values (hours with 100 steps or more). This approach allowed us to have a better understanding of the participants’ physical activity patterns and to compare them between the intervention and the control group, but it could also have influenced the outcome results. In designing a future study, we should consider adopting a study protocol that establishes a better compliance with wearing the sensors and monitors the wearing of the sensors to enable us to recognize the activities better and to ensure reliable data acquisition. Another approach would be changing the activity tracker system to a bracelet or a sensor that is embedded in clothing that can provide 24 h wearing or providing multiple trackers to each participant to achieve higher compliance in wearing.

The physical activity results were captured for 9 weeks. The data for the control group show that the physical activity decreased during weeks 4, 7 and 9 comparing to the baseline week. This may contribute to the results observed in the study by Tabak [10], in which a decrease in the physical activity of the participants during a physical activity intervention occurred after 4 weeks. For the intervention groups, we did not observe a decrease in physical activity; however, a considerable increase compared to the baseline week was observed at week 9. This could indicate a positive effect of a virtual community and in this case due to the sharing of physical activity data, the competition element between groups and collaboration between peers. The length of the intervention can have an impact on the results since adopting, adapting and adhering to one or more lifelong lifestyle changes takes time before the new lifestyle can be considered established (approximately 6 months as stated by the transtheroretical model).

Additionally, we were not able to investigate the relationship between the obtained results and the participants’ profiles (habitual physical indexes (work, sport and leisure indexes), quality of life, BMI and stage of change). According to the intervention group’ results, we were able to see a relationship between being physically active during leisure time (through reported leisure index) and average number of steps per week, and between being physically active during leisure time accessing the system. Although it was a negative association, this can contributes to the influence of the system on the intervention group comparing to the control group. The fact that participants reported their habitual physical indexes could have an influence on the results. In a later study, the relationships should be investigated in order to confirm the finding.

This study was not an effectiveness study. TogetherActive V2 is in a relatively early stage of development (stage I according the evaluation framework proposed by deChant [41]). We aimed in this study to investigate and explore the outcome of predefined parameters and to address these outcomes for a virtual community (the intervention group) comparing to a system without a virtual community (the control group). Additional factors and parameters in the study protocol should be considered when designing an effectiveness study, e.g., the target population and its properties (habitual physical indexes, the quality of life, BMI and stage of change), the length of the intervention and the total number of participants (which would fall into stage II in the evaluation framework proposed by deChant [41]). Based on the reported habitual physical (work, sport and leisure) indexes, the quality of life and BMI index values show that most of these participants experienced similar lifestyle and health conditions. With respect to their state of change, the participants were spread out over the 5 different stages of change. The results could be influenced by heterogeneous sample regarding their stage of change. Additionally, the length of the intervention and the number of participants (thus the amount of collected data) were limited. In a later study, the composition of the participants based on their profile, length of the study and number of participants should be investigated.

In this exploratory study, only healthy participants were included. A system such as TogetherActive V2 may prove to be beneficial for target groups with specific chronic conditions, hence building a community of people with this condition. It may also prove to be beneficial for target groups with similar states of change, particularly for the action and the maintenance states. We propose that in future studies a different user group should be targeted. Given the increased awareness and evidence regarding the importance of physical activity for prevention and treatment, one possible target group is persons with a chronic condition (e.g., obesity, diabetes, osteoporosis, cardiovascular disease and cancer) [1, 42, 43]. Another target group could be elderly people. Research has shown that many elderly experience loneliness [44] and that their physical activity levels are influenced by their loneliness [45].

## Conclusions

This study is an exploratory study that addresses the potential differences between a virtual community coupled with a physical activity monitoring system and a traditional physical activity system.

This study has shown one potential difference in having a later decrease in physical activity level for the virtual community and no considerable difference in the actual amount of physical activity or in the degree of adherence to the goal of 10,000 steps per day. Some difficulties were encountered in the analysis of the resulting steps data to be able to draw the right conclusion about activities. We were able to overcome these difficulties by finding a useful method that addressed time spent wearing or not wearing the sensor and by defining hours with no activity (HNA), hours with low activity (HLA) and hours with activity (HA).

Compared to our first study [11], we solved problems with the size and battery life of the activity tracker system that had previously presented inconveniences in wearing and using it daily. However, with the new activity tracker system, Fitbit, we wear not able to control the participants’ wearing behaviour or the quality of the obtained data. As a challenge for future work, it is necessary to develop a required functionality in the system that can monitor the wearing of the sensor and make it possible to interact with participants to ensure wearing the sensor. This would increase the reliability of the resulting physical activity data. This could also be overcome by changing the activity tracker system into a bracelet or an embedded sensor in clothing that can provide 24 h of use or by providing multiple trackers to one person to assure higher compliance in wearing. It is also possible to consider including in the protocol sport training for the participants, which may increase their motivation and adherence.

The current TogetherActive V2 design does not include physical activity activation concepts; hence, it does not offer feedback or feedback modalities designed to initiate physical activity. Such approaches have already been investigated in, for instance, [46]. A future development we would like to explore is the use of social activation with involvement of peers to enhance the communication between peers and to improve their physical activity levels. Future evaluations will include healthy subjects, and we will investigate the added value of virtual community in improving physical activity levels. However, the concepts presented in this paper can easily be transferred to other application domains, including the lifestyle change support that is needed by many chronic patients and in the domain of prevention, especially within identified groups with increased risk.

Setting up a larger follow-up study with a larger number of participants and a greater resulting amount of data will make it possible to investigate relationships between using virtual communities and the physical activity levels. The results of this follow-up study would provide additional input that could be considered in an effectiveness study of the virtual community on physical activity.

## Footnotes

1
https://www.randomlists.com/team-generator

2 Institute for Biomedical Technology and Technical Medicine https://www.utwente.nl/mira/

3 Centre for Telematics and Information Technology https://www.utwente.nl/ctit/
